# Harmonisation of Low-Density Lipoprotein Cholesterol Results Obtained with Different Direct Methods: A Study Based on an External Quality Assessment Program

**DOI:** 10.3390/jcm14248706

**Published:** 2025-12-09

**Authors:** Agnieszka Ćwiklińska, Aleksandra Fijałkowska

**Affiliations:** 1 Department of Clinical Chemistry, Medical University of Gdańsk, Dębinki 7, 80-211 Gdańsk, Poland; 2 Labquality Polska Sp. z o.o., Parkowa 6/1 C, 81-549 Gdynia, Poland; aleksandra.fijalkowska@aurevia.com

**Keywords:** direct method, harmonisation, LDL-C

## Abstract

**Objectives:** Low-density lipoprotein cholesterol (LDL-C) is a primary lipid cardiovascular risk factor, for which universal decision cut-off values are applied. The aim of our study was to assess the current harmonisation status of LDL-C results obtained with direct methods. **Methods:** We analysed LDL-C results obtained in an international external quality assessment (EQA) programme ‘Lipids and Lipoproteins, Lp(a)’ within the period of January 2020 to October 2025. The samples were fresh liquid unprocessed human sera obtained from single donors. The inter-laboratory coefficient of variation (CV), the intra-method CV, and the concentration difference (mmol/L) between the method-specific mean and consensus LDL-C values were analysed. **Results:** The median of inter-laboratory CV was 8.7% (Q1–Q3: 7.3%–9.9%). The medians of intra-method CV were from 3.3% (Q1–Q3: 2.6%–4.0%) for ‘Roche’ to 8.6% (4.9%–13.7%) for ‘Siemens (Advia & Attelica)’. The median concentration of LDL-C for individual method groups was as follows: 3.45 (Q1–Q3: 2.47–4.59) mmol/L for ‘Abbott’, 3.31 (2.15–4.11) mmol/L for ‘Roche’, 3.50 (2.02–4.47) mmol/L for ‘Siemens (Advia & Attellica)’, and 3.25 (2.27–4.19) mmol/L for ‘Thermo Scientific’. The greatest differences between the method groups were observed for serum samples with high LDL-C and high triglyceride levels, for which the medians of concentration difference between the method-specific mean and consensus LDL-C values were from −0.20 (−0.37–(−0.13)) mmol/L to 0.39 (0.30–0.52) mmol/L. **Conclusions:** Harmonisation of LDL-C results remains a challenge. Healthcare professionals and patients should be aware of possible differences between LDL-C results obtained from different analysers/manufacturers.

## 1. Introduction

Low-density lipoprotein cholesterol (LDL-C) is identified as a major lipid cardiovascular disease (CVD) risk factor and its measurement is recommended as the primary lipid analysis for screening, diagnosis, and management of CVD [1]. In routine laboratories, LDL-C is calculated using mathematical formulas, most often the Friedewald formula, or measured using direct homogeneous methods [1].

LDL-C calculation with the Friedewald formula is a widespread method that is convenient and cost-effective, but it has some important limitations such as the underestimation of LDL-C results in samples with high triglyceride (TG) or low LDL-C concentrations [2]. Given limitations of the Friedewald equation, alternative equations have been developed, from which the Martin–Hopkins and the Sampson–NIH formulas are the most popular [3]. Also, the LDL-C direct homogeneous methods are widely used in laboratories, especially for hypertriglyceridemic samples [3]. These methods allow direct determination of cholesterol in LDL particles with the use of enzymatic techniques, after solubilisation or blocking other lipoprotein fractions [4].

Homogeneous LDL-C methods are fully automated and easy to use, and they are available from various manufacturers that differ in the applied reagents, enzymes, and the methodology of separation of LDL from other lipoproteins [5]. It has been shown that the differences between manufacturers can result in differences between the methods in terms of their accuracy and precision [6].

Since LDL-C is a laboratory parameter for which universal decision cut-off values are applied, the harmonisation of LDL-C results determined with different methods is a very important contributor essential for the correct classification of patients into appropriate CVD risk groups and their treatment [1,7].

Using data of an international external quality assessment (EQA) programme, we aimed to assess the current harmonisation of LDL-C results obtained with different methods available in routine practice. 

## 2. Materials and Methods

### 2.1. Scheme Organisation

LDL-C results obtained in the EQA scheme ‘Lipids and Lipoproteins, Lp(a)’ provided by Aurevia, Finland (formerly Labquality, Helsinki, Finland) were analysed. Labquality is an independent EQA provider with over 50 years of experience. The scheme ‘Lipids and Lipoproteins, Lp(a)’ is an accredited scheme (ISO/EN 17043) [8], in which laboratories from 13 to 18 (depending on a survey) different countries participate. The scheme is organised four times a year (it was organised twice a year until 2022), and two serum samples in each survey are sent to laboratories for lipid profile analysis, including LDL-C. The samples are fresh liquid unprocessed human sera obtained from single donors. Each laboratory participating in a survey performs one LDL-C determination for each sample. Laboratories’ results obtained in a survey are classified into the method groups that are direct LDL-C determination method groups established according to the analysers’ manufacturer (except the ‘Friedewald’ group).

### 2.2. Analysis of the Results

**Figure 1:**
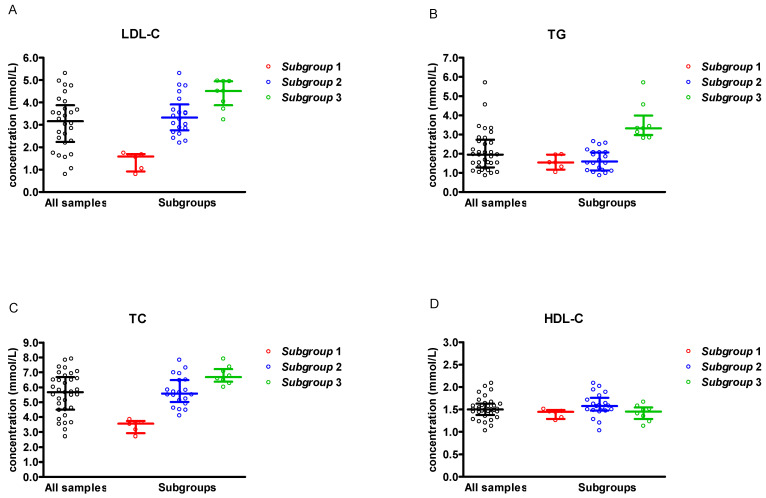
Lipid profile results obtained in the scheme ‘Lipids and Lipoproteins, Lp(a)’ within the period of January 2020–October 2025 (*n* = 34). (**A**) LDL-C; (**B**) triglycerides (TG); (**C**) total cholesterol (TC); (**D**) HDL-C. Each point indicates the mean of the laboratories’ results for individual samples. The line presents median and interquartile range. Subgroup 1—samples with LDL-C < 1.8 mmol/L and TG < 2.75 mmol/L; Subgroup 2—samples with LDL-C ≥ 1.8 mmol/L and TG < 2.75 mmol/L; Subgroup 3—samples with LDL-C ≥ 1.8 mmol/L and TG ≥ 2.75 mmol/L. Black circles represent ‘all samples’ that is presented on OX axis.

In total, 1495 laboratories’ results obtained for 34 serum samples in 17 surveys performed from January 2020 to October 2025 were analysed. The LDL-C concentration and the concentration of other lipid profile parameters for the analysed samples are presented in Table 1 and Figure 1.

For each sample in a survey, the inter-laboratory coefficient of variation (CV) was calculated. For each method group, the intra-method CV and the concentration difference (mmol/L) between the method-specific LDL-C mean and consensus LDL-C values were calculated. The consensus LDL-C value was determined as the median of the method-specific LDL-C mean values.

In the analyses according to the method group, the following method groups were evaluated: ‘Abbott’ (*n* = 330), ‘Roche’ (*n* = 605), ‘Siemens (Advia & Atellica)’ (‘Siemens Advia’ until 1-2022 survey) (*n* = 231), and ‘Thermo Scientific’ (*n* = 152). From this analysis, 12% of all laboratories’ results have been excluded, that of which was obtained from the method groups not listed above, with a very low number of laboratories (i.e., with the mean number of results per sample below 2). It was as follows: ‘Beckman Coulter’ (*n* = 62), ‘Diasys’ (*n* = 4), ‘Friedewald’ (*n* = 33), ‘NMR spectroscopy’ (*n* = 42), ‘Siemens’ (without Advia and Atellica) (*n* = 30), and ‘Vitros’ (*n* = 6).

The analyses were performed for all samples and in the subgroups, taking into account LDL-C and triglyceride (TG) concentrations in the serum samples. For analysis according to lipid concentration, the samples were divided into three subgroups. Subgroup 1 included samples with low LDL-C, below 1.8 mmol/L (TG for these samples was below 2.75 mmol/L). Subgroup 2 and Subgroup 3 included samples with LDL-C ≥ 1.8 mmol/L and differed in the TG level. Subgroup 2 had TG level < 2.75 mmol/L, and the Subgroup 3 had TG ≥ 2.75 mmol/L (Table 1, Figure 1).

Statistical analysis was performed using GraphPad Prism 5 software (GraphPad Software, San Diego, CA, USA). The normality of data distribution was assessed using the Shapiro–Wilk test. The data is presented as mean ± standard deviation (SD) or median and interquartile range (Q1–Q3). The difference between method groups was assessed using Kruskal–Wallis test or Repeated Measures ANOVA with Tukey’s Multiple Comparison Test as post hoc test. The statistical significance was established as *p* < 0.05.

## 3. Results

**Figure 2:**
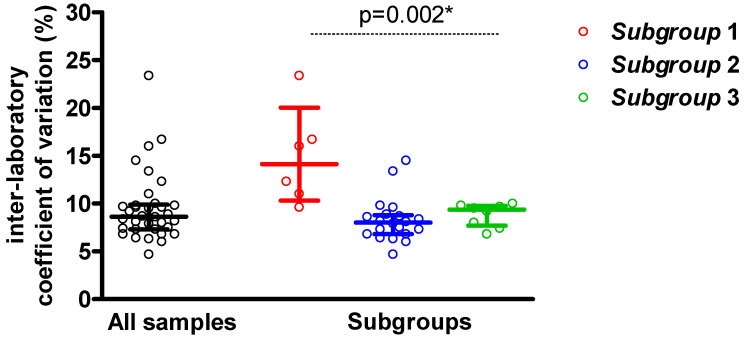
Inter-laboratory coefficient of variation (%) of LDL-C results. Each point indicates inter-laboratory coefficient of variation (%) for individual sample. The line presents median and interquartile range. Subgroup 1—samples with LDL-C < 1.8 mmol/L and TG < 2.75 mmol/L; Subgroup 2—samples with LDL-C ≥ 1.8 mmol/L and TG < 2.75 mmol/L; Subgroup 3—samples with LDL-C ≥ 1.8 mmol/L and TG ≥ 2.75 mmol/L. * Kruskal–Wallis test. Black circles represent ‘all samples’ that is presented on OX axis.

For all analysed samples, the median of inter-laboratory CV was 8.7% (Q1–Q3: 7.3%–9.9%). The highest median of inter-laboratory CV was for the Subgroup 1: 14.2% (10.3%–20.1%). For the Subgroup 2, it was 8.0% (6.8%–8.8%), and for the Subgroup 3, it was 9.4% (7.7%–9.8%) (Figure 2).

**Figure 3:**
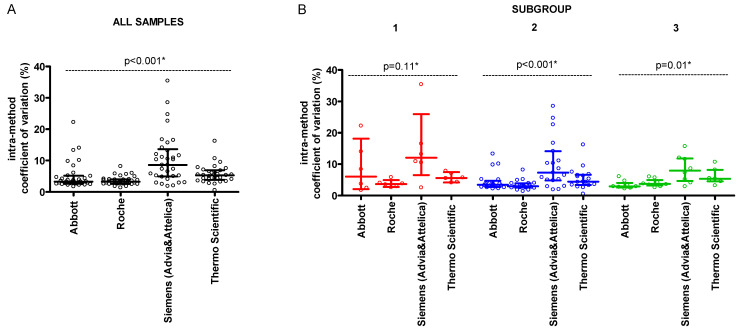
Intra-method coefficient of variation (%) of LDL-C results. (**A**) intra-method coefficient of variation (%) for all analysed samples (*n* = 34); (**B**) intra-method coefficient of variation (%) according to the subgroups. The line presents median and interquartile range. Each point indicates intra-method coefficient of variation (%) for individual samples. Subgroup 1—samples with LDL-C < 1.8 mmol/L and TG < 2.75 mmol/L; Subgroup 2—samples with LDL-C ≥ 1.8 mmol/L and TG < 2.75 mmol/L; Subgroup 3—samples with LDL-C ≥ 1.8 mmol/L and TG ≥ 2.75 mmol/L. * Kruskal–Wallis test.

For individual method groups, the medians of intra-method CV were from 3.3% (Q1–Q3: 2.6%–4.0%) for ‘Roche’ to 8.6% (4.9%–13.7%) for ‘Siemens (Advia & Attelica)’ (Figure 3A). For none of the method groups, the medians of intra-method CV differed significantly between the subgroups with different lipids level (Figure 3B).

**Figure 4:**
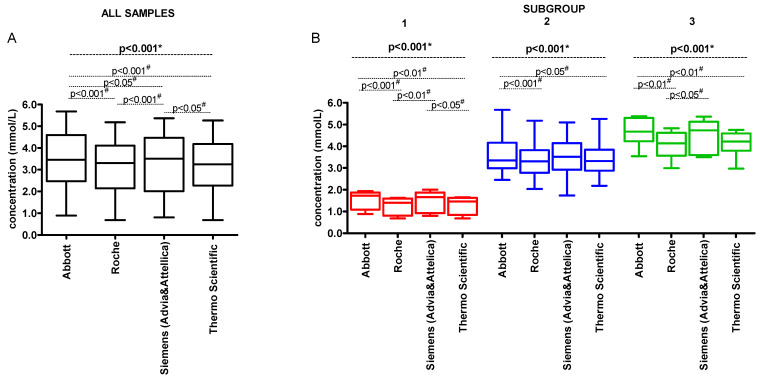
The LDL-C concentration (mmol/L) according to the method groups. (**A**) the LDL-C concentration (mmol/L) for all analysed samples; (**B**) the LDL-C concentration (mmol/L) according to the subgroups. Data are presented as median, interquartile range, and minimum and maximum. Subgroup 1—samples with LDL-C < 1.8 mmol/L and TG < 2.75 mmol/L; Subgroup 2—samples with LDL-C ≥ 1.8 mmol/L and TG < 2.75 mmol/L; Subgroup 3—samples with LDL-C ≥ 1.8 mmol/L and TG ≥ 2.75 mmol/L. * Repeated Measures ANOVA; # Tukey’s Multiple Comparison Test (post hoc).

The median concentration of LDL-C for individual method groups was as follows: 3.45 (Q1–Q3: 2.47–4.59) mmol/L for ‘Abbott’, 3.31 (2.14–4.11) mmol/L for ‘Roche’, 3.50 (2.01–4.47) mmol/L for ‘Siemens (Advia & Attellica’, and 3.25 (2.27–4.19) mmol/L for ‘Thermo Scientific’ (Figure 4A). For the Subgroup 1, the median concentration of LDL-C varied from 1.41 (0.81–1.60) mmol/L for ‘Roche’ to 1.73 (1.09–1.87) mmol/L for ‘Abbott’. For the Subgroup 2 it was from 3.31 (2.78–3.83) mmol/L for ‘Roche’ to 3.52 (2.92–4.14) for ‘Siemens (Advia & Attellica)’. For the Subgroup 3 the lowest median value of 4.14 (3.57–4.62) mmol/L was obtained for ‘Roche’ and the highest median value of 4.73 (3.60–5.13) mmol/L was obtained for ‘Siemens (Advia & Attellica)’ (Figure 4B).

**Figure 5:**
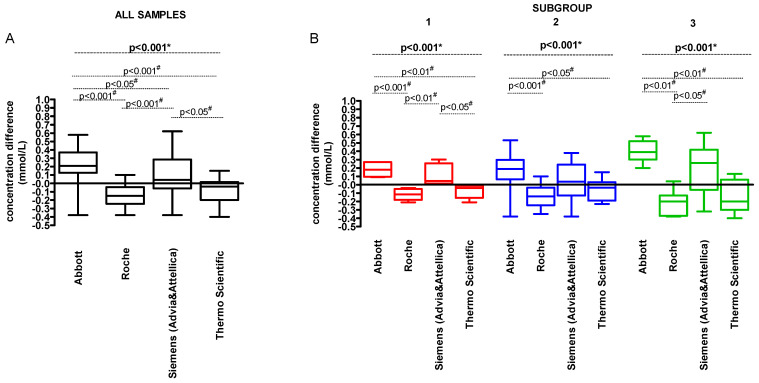
The concentration difference (mmol/L) between the method-specific LDL-C and consensus LDL-C values. (**A**) the concentration difference (mmol/L) for all analysed samples; (**B**) the concentration difference (mmol/L) according to the subgroups. The line presents median and interquartile range. Each point indicates concentration difference for individual sample. Subgroup 1—samples with LDL-C < 1.8 mmol/L and TG < 2.75 mmol/L; Subgroup 2—samples with LDL-C ≥ 1.8 mmol/L and TG < 2.75 mmol/L; Subgroup 3—samples with LDL-C ≥ 1.8 mmol/L and TG ≥ 2.75 mmol/L. * Repeated Measures ANOVA; # Tukey’s Multiple Comparison Test (post hoc).

The median of difference between the method-specific and consensus LDL-C value varied from −0.15 (Q1–Q3: −0.25–(−0.05) mmol/L) for ‘Roche’ to 0.21 (0.13–0.37) mmol/L for ‘Abbott’ (Figure 5A). For the Subgroup 1, the median of difference between the method-specific and consensus LDL-C values varied from −0.12 (Q1–Q3: −0.18–(−0.05) mmol/L) for ‘Roche’ to 0.18 (0.10–0.27) mmol/L for ‘Abbott’ method group. For the Subgroup 2, it was from −0.14 (−0.25–(−0.04)) mmol/L for ‘Roche’ to 0.19 (0.07–0.30) mmol/L for ‘Abbott’. For the Subgroup 3 the range of differences was the widest and it was from −0.20 (−0.37–(−0.13)) mmol/L for ‘Roche’ to 0.39 (0.30–0.52) mmol/L for ‘Abbott’ (Figure 5B).

## 4. Discussion

In our study based on the EQA programme, we evaluated the harmonisation of LDL-C results obtained with different direct methods.

The guidelines for the management of dyslipidemia allows LDL-C to be either calculated or measured directly, and homogeneous methods are widely available in routine laboratories as an alternative for LDL-C mathematical formulas [1]. Moreover, the definition of LDL-C in direct and indirect methods is the same, representing the sum of cholesterol in LDL, intermediate-density lipoprotein (IDL), and lipoprotein (a) [1]. Thus, one would expect at least a good agreement between the methods, and the more, among direct assays.

For the general population, there is a strong correlation between calculated and measured LDL-C [9], and due to well-known limitations of the Friedewald equation, there is a high awareness of the possibility of obtaining underestimated LDL-C results calculated in patients with hypertriglyceridemia or low LDL-C concentration [1]. However, there could be a lack of awareness of the limitations of direct methods and the problem of discrepancies in results obtained with these methods.

LDL is a heterogeneous group of lipoproteins differing in size, density and composition [1] and it has been observed that different LDL-C direct assays can be more or less sensitive for LDL subclasses, and also for IDL particles, both included, according to definition, to LDL-C [10]. Moreover, other lipoprotein particles such as TG-rich chylomicron and very-low-density lipoprotein (VLDL), and abnormal lipoprotein such as lipoprotein X can react with LDL-C reagents [11,12,13]. It has also been shown that an extreme high or low concentration of high-density lipoprotein (HDL) can be a source of differences between commercially available LDL-C homogeneous assays [14].

In a study of seven LDL-C direct methods conducted by Miller et al., the bias for different direct methods assessed in relation to the reference ultracentrifugation procedure ranged from −6.8% to +1.1% in healthy individuals, and from −11.8% to 4.1% in ’diseased’ subjects (i.e., individuals with known CVD or dyslipidemias) [6]. In a study based on the EQA programme, in which the results obtained in one survey from ~50 laboratories were assessed, the challenges of precise and accurate LDL-C measurement were also highlighted, especially for hypertriglyceridemic samples [15]. We observed that the range of concentration differences between the method-specific and consensus LDL-C values was the widest (up to 0.59 mmol/L) for the group including hypertriglyceridemic samples. The observed differences were most probably related to the increased presence of TG-rich lipoproteins: VLDL and/or lipoprotein remnants in these samples, cholesterol of which could be the substrate for some LDL-C reagents. Liang et al. also observed that homogeneous LDL-C assays showed TG-dependent variability and concluded that these discrepancies were likely attributable to divergent reagent formulations and reaction principles [16]. Kwon et al. also observed, in their recent study, that remnant cholesterol can serve as a significant source of positive bias in direct LDL-C methods, which vary according to the measurement system [17]. This can confirm that in individual method groups, the reactivity with LDL particles can be different and/or the non-specific reactions can occur, especially in the samples with high TG concentration containing the increased number of those other than LDL lipoprotein particles.

Our study has some limitations. Since LDL-C results from reference ultracentrifugation procedure were not available, method-specific LDL-C mean values were compared to LDL-C median consensus values [18]. It should also be noted that method groups differed in terms of the total number of laboratories and, in some surveys, there was a low number of laboratories in individual method groups; that could affect the reliability of the results in the individual surveys. It should also be considered that method groups included various analysers that could affect the intra-method variability. The impact of the analyser on the within-method dispersion of the results can be observed for ‘Siemens (Advia&Attelica)’ group. For the surveys 1-2020—1-2022 in which only Advia analysers were included to the group, the median intra-method CV was 3.6% and it was significantly lower than for all analysed surveys. Moreover, the composition and lot numbers of the reagents, and the information if closed- or open-channel reagents were used are not provided by the EQA survey participants. Thus, the impact of these factors on the variability of the results could not be evaluated. An interesting would also be to analyse the impact of the clinical status of the donors, but such data were not available in the EQA scheme. Thus, we grouped and analysed the obtained results according to the measured lipid parameters. An independent assessment of the quality of measurements in laboratories under routine conditions performed with the EQA programme is a very important approach to obtain harmonised laboratory results, and commutability of control materials is an essential issue to be able to reliably assess the quality of the results [18,19]. The results of the EQA programme analysed in this study were obtained for fresh liquid unprocessed human sera from single donors that should be commutable [20] but, unfortunately, we were not able to assess this issue. However, the results of our study are in line with other studies performed with the use of native and control samples. For instance, Miller et al. observed consistent negative bias for Roche reagents [6]. Langlois et al. also showed a negative mean bias for Roche and positive mean bias for Abbott [15]; that is in line with the results of our study. Liang et al. observed that Roche exhibited superior robustness on hypertriglyceridemia, up to TG 16.9 mmol/L [16]. In our study, for this method group, we observed the smallest fluctuations in the concentration differences between the subgroups with different lipid levels. Thus, taking into account the type of sample and the fact that our results are in line with previous studies we conclude that findings of our study were not significantly affected by the commutability of the samples.

In conclusion, to obtain a clinically valid result, a test should selectively, accurately, and precisely measure the marker of interest. LDL is a heterogeneous group of lipoproteins differing in size, density, and composition, and LDL-C direct assays are based on different principles. Thus, LDL-C accurate determination is a big challenge, especially in the presence of increased number of other lipoproteins in dyslipidemic and hyperlipidemic samples, for which the non-specific reactions can occur. Test manufacturers should be aware of the limitations of the methods and further efforts to improve the standardisation of LDL-C measurements are needed. Moreover, the current recommended LDL-C treatment goals are independent from methods used in the laboratory; thus, non-harmonisation of LDL-C results can represent a concrete risk for patient management. Healthcare professionals and patients should be aware of possible differences between LDL-C results obtained with different analysers/manufacturers.

## Figures and Tables

**Table 1:** Lipid profile results obtained in the scheme ‘Lipids and Lipoproteins, Lp(a)’ within the period of January 2020–October 2025.

	Parameter	Concentration (mmol/L)
		Mean ± SD (Minimum–Maximum)
All serum samples (*n* = 34)	LDL-C	3.29 ± 1.22 (0.79–5.31)
	TG	2.12 ± 1.07 (0.86–5.70)
	TC	5.57 ± 1.39 (2.70–7.91)
	HDL-C	1.52 ± 0.24 (1.03–2.09)
Subgroup 1 (*n* = 6)	LDL-C	1.41 ± 0.39 (0.79–1.74)
	TG	1.55 ± 0.36 (1.03–1.96)
	TC	3.41 ± 0.41 (2.70–3.85)
	HDL-C	1.41 ± 0.10 (1.26–1.51)
Subgroup 2 (*n* = 20)	LDL-C	3.42 ± 0.89 (2.19–5.31)
	TG	1.68 ± 0.56 (0.86–2.64)
	TC	5.72 ± 1.01 (4.10–7.83)
	HDL-C	1.60 ± 0.27 (1.03–2.09)
Subgroup 3 (*n* = 8)	LDL-C	4.36 ± 0.65 (3.23–4.97)
	TG	3.63 ± 1.00 (2.81–5.70)
	TC	6.81 ± 0.62 (6.02–7.91)
	HDL-C	1.42 ± 0.18 (1.13–1.67)

LDL-C—cholesterol LDL; TG—triglycerides; TC—total cholesterol; HDL-C—cholesterol HDL. Subgroup 1—samples with LDL-C < 1.8 mmol/L and TG < 2.75 mmol/L; Subgroup 2—samples with LDL-C ≥ 1.8 mmol/L and TG < 2.75 mmol/L; Subgroup 3—samples with LDL-C ≥ 1.8 mmol/L and TG ≥ 2.75 mmol/L.

## Data Availability

The raw data supporting the conclusions of this article will be made available by the authors on request.

## Abbreviations

The following abbreviations are used in this manuscript:

CV	Coefficient of variation
CVD	Cardiovascular disease
HDL-C	High-density lipoprotein cholesterol
EQA	External quality assessment
IDL	Intermediate-density lipoprotein
LDL-C	Low-density lipoprotein cholesterol
SD	Standard deviation
TC	Total cholesterol
TG	Triglycerides
VLDL	Very-low-density lipoprotein
Q	Quartile
